# Use of grape pomaces to produce biomass of a*Komagataella pastoris* strain expressing a bovine chymosin activity

**DOI:** 10.1002/fsn3.128

**Published:** 2014-09-12

**Authors:** Diego Kingston, Guido F Novelli, Patricia Cerrutti, Matias N Recupero, Martin Blasco, Miguel A Galvagno

**Affiliations:** 1 Departamento de Ingeniería, Química Facultad de Ingeniería, Universidad de Buenos Aires, Pabellón de Industrias, Ciudad UniversitariaIntendente Güiraldes 2160 (1428), Buenos Aires, Argentina; 2 IIB-INTECH-UNSAM-CONICET25 de Mayo y Francia, (1650), San Martín, Buenos Aires, Argentina

**Keywords:** Agroindustrial by-product, bovine chymosin, *Komagataella pastoris* biomass

## Abstract

The use of agroindustrial wastes not only decreases bioprocesses and disposal costs but also contributes to the upgrading of the residues. An active recombinant methanol-inducible bovine chymosin has been expressed in our laboratory in the yeast*Komagataella pastoris*, and grape pomace extracts (GRE) were proposed as a convenient C-energy source for the biomass production of the genetically engineered strain. Carbon and nitrogen sources, growth factors, and initial pH conditions were selected by classical methodology; thereafter, growth conditions optimization was performed using statistical designed experiments (DoEs). In the presence of (in g·L^−1^) 67.0 monosaccharides (glucose and fructose) from GRE, 5.0 (NH_4_)_2_SO_4_, and 10.0 sugar cane molasses (CMz), a yield of 20.0 g·L^−1^ cell dry weight (CDW) was obtained aerobically after 60 h incubation at 28°C and pH 4.0. Applying a fed-batch strategy with methanol:sorbitol as the enzyme inducers, a chymosin production of 8.53 International Milk Clotting Units (IMCU) per mg protein was obtained in the supernatant. The results presented show that through a statistical design, a simple, cheap, and easy to prepare culture medium could be developed using two agroindustrial derivatives (GRE and CMz) to obtain a higher value added product.

## Introduction

Chymosin (rennet, E.C. 3.4.23.4) is an aspartic protease used for milk clotting in the first step of cheesemaking. This enzyme specifically breaks down the Phe105-Met106 peptide bond of milk*κ*-casein to form insoluble para-*κ*-casein (Mohanty et al. 1999), contributing to micelle precipitation in the presence of calcium ions. Coagulants used for cheese manufacturing come from different sources, such as microorganisms, plants, animals, and recombinant protein expression, and each source has different characteristics determining their applications (Jiang et al. 2012). The methylotrophic yeast*Komagataella* (*Pichia*)*pastoris* is extensively used for the expression of heterologous proteins (Sreekrishna and Kropp 1996) because it exhibits several advantages over other microbial systems like*Escherichia coli*: rapid growth and high-density fermentations (Cregg et al. 2009) and the ability to perform many of the eukaryotic posttranslational protein modifications like folding, proteolytic processing, glycosylation, and disulfide bond formation (Cereghino et al. 2002).

Several milk coagulants from various mammalian sources have been efficiently expressed in*K. pastoris*, such as buffalo (Vallejo et al. 2008), bovine (constitutive expression) (Jiang et al. 2012), and goat chymosins (Vallejo et al. 2012). A typical fermentation process for production of biomass and for an inducible recombinant protein expression in*K. pastoris* using Mut^+^ and/or Mut^−^ strains (Zhang et al. 2000) can be broadly divided into two to three steps. First, a batch-growing phase in a medium containing either a fermentable sugar or glycerol to produce biomass, generally followed by an optional fed-batch transition phase on glycerol. Finally, to obtain the desired product, an induction fed-batch phase on methanol is performed (Sreekrishna and Kropp 1996).

High-microbial-cell concentrations are needed to achieve high recombinant protein yields. A basal salt medium (BSM) proposed by Invitrogen (Invitrogen Argentina S.A., Buenos Aires, Argentina) is commonly employed to attain the high-density cultures, but it is not always the ideal supplement due to its high ionic strength, medium components precipitation, and imbalanced nutrient concentrations (Cos et al. 2006). Moreover, the media is difficult to prepare for large scale uses and requires the addition of biotin which is an expensive vitamin, and of a mixture of micronutrients, many of which may inhibit recombinant chymosin production in this strain (Jiang et al. 2012). Thus, there is an actual demand for formulation and optimization of an alternative growth medium.

Cost reduction in bioprocesses is crucial for the expansion of a biotechnological product market obtained by microbiological fermentation. Besides, optimization of the fermentation medium is included in the strategic analysis of the viability of any biotechnological process. Economic evaluation of microbial biomass production has suggested that raw materials costs (mainly the C source) in fermentations could attain up to 60% of the total manufacturing cost (Vogel and Todaro 1997). Therefore, it is desirable to produce biomass from cheap substrates or even from by-products as is the case of wine-making residues (Vazquez et al. 2013).

Agricultural wastes that are a huge environmental problem, can represent a source for the production of high value added products (Rebecchi et al. 2013). Grape pomace, the residue left after juice extraction, is the most abundant wine-making waste representing about the 20% (w/w) of grapes used for the production of wine. It is rich in carbohydrates although its protein content is rather low. The challenge is the utilization of this by-product in fermentation processes, even though nutritional supplementations were required, to obtain high commercial value products, instead of its disposal to the environment (Hang et al. 1986; Schieber et al. 2001).

Optimization of culture conditions can be conducted by changing one factor at a time or more efficiently by varying several simultaneously, and statistically examining their linear and quadratic effects. Statistical data analysis also enables visualization of the interactions between the experimental variables, revealing data predictions that are not directly covered by experimentation (Kalil et al. 2000; Mandenius and Brundin 2008). Thus, DoEs is an organized approach that yields more reliable information*per* experiment than classical approaches. However, the current literature contains scanty information of DoEs for bioprocesses involving*K. pastoris*. For example, Ghosalkar et al. (2008), Holmes et al. (2009), and Lin et al. (2007) used DoEs for optimizing specific yield in the induction phase of a recombinant protein produced by this yeast species.

The aim of this study was to optimize biomass production of a recombinant*K. pastoris* strain constructed in our laboratory, transformed with a bovine prochymosin sequence and secreting an inducible active chymosin activity to the fermentation medium. Specifically, we have explored the possibility of using by-products from wine production as the main component of a simple and low-cost growth medium. To achieve these objectives, systematic and statistical optimization studies were carried out.

## Materials and Methods

### Construction of a recombinant*Komagataella pastoris* strain

The construction and selection of a recombinant*Komagataella* (*=Pichia*)*pastoris* strain (Clone 1) by transforming the strain GS115 (Invitrogen), expressing inducible bovine chymosin activity, was performed in our laboratory as described in Noseda et al. (2013).

### Media and culture conditions

Along this work, the following media and media components were employed as indicated in each case: YPD agar medium containing 10 g·L^−1^ yeast extract (YE), 20 g·L^−1^ meat peptone, 20 g·L^−1^ glucose, and 20 g·L^−1^ agar–agar; Yeast Nitrogen Base (YNB) (Difco™ Ref. 239210; Becton, Dickinson and Company, Sparks, MA) and YNB without amino acids and ammonium sulfate (YNB w/o; Difco™, Ref 233520). BSM and PTM1 solution (Sreekrishna and Kropp 1996) were also used.

Solutions of YE, YNB, and YNB w/o, biotin, methanol, urea, and BSM were filter sterilized (0.22 *μ*m cellulose acetate membrane) and aseptically added to other media components previously sterilized at 121°C for 15 min.

The seeds and stems of pomace from*Semillon* grapes pressed for wine making were separated. The remaining raw material (skins and residual pulp) was stored at −20°C until used. The grape residues were washed three times with distilled water and then homogenized in a Waring blender using a minimum required volume of 0.10 mol/L citric acid–sodium citrate buffer (CCB) at pH = 4.0–5.0. The extract obtained (GRE) was filtered through cheesecloth and centrifuged (5000*g* for 5 min at 4°C) to separate solids and the clarified liquid filtrate was frozen at −20°C until used. Reducing sugar content (RS) in GRE, identified as d-glucose plus d-fructose, was 67.0 g·L^−1^.

Crude glycerol derived from biodiesel production was treated as described by Chi et al. (2007). The content of authentic glycerol after this purification step was 601.0 g·L^−1^.

Sugarcane molasses (CMz) containing 508.0 g·kg^−1^ fermentable sugars was used. All other chemicals were of analytical grade or of the highest purity available, and used without further purification.

Clone 1 cells were maintained in agar YPD slants at 4°C for routine assays. For inoculum preparation, except when indicated, a loopful of the culture was transferred to 20-mL YPD broth and incubated for 24 h at 28°C. Then, cells were harvested by centrifugation (5500*g* for 15 min) and pellets were washed three times with distilled water prior to their inoculation in the experimental flasks at an initial cell concentration of OD_600nm_ = 0.15 units.

### Effect of carbon sources

Experiments to evaluate the C sources effects on the yeast growth, were performed in a complete yeast growth medium as YNB, supplemented with analytical glycerol (25.0, 50.0, and 100.0 g·L^−1^), treated glycerol derived from biodiesel (25.0 g·L^−1^), d-glucose (40.0 g·L^−1^), or GRE (37.5 g·L^−1^ RS).

### Effect of nitrogen sources

Experiments were carried out in YNB w/o media using GRE (67 g·L^−1^) as source of RS and supplemented with the following organic or inorganic N sources in order to attain a total N concentration = 2.5 g·L^−1^ and a C:N ratio = 10:1: meat peptone, tryptone, (NH_4_)_2_SO_4_, corn steep liquor (CSL), and urea. The initial pH value was fixed at 4.8–5.0 units with 0.10 mol/L CCB.

### Effect of pH

The growth media containing GRE were adjusted to pH 3.0, 4.0, 5.0, or 6.0 with 0.10 mol/L CCB. Then, a 10-fold concentrated YNB solution was added aseptically to each GRE solution at the different adjusted pH values.

### Effect of vitamins

The influence of vitamin addition to GRE was studied in media containing GRE and 5.0 g·L^−1^ (NH_4_)_2_SO_4_ at pH = 5.0 (0.10 mol/L CCB) supplemented with 500 *μ*g·L^−1^ biotin, 10.0 g·L^−1^ CMz, or 3 g·L^−1^ YE.

### DoEs

Plackett–Burman and Full Fractional Factorial screening designs (PBSD and FFSD) were applied to screen culture variables that may significantly affect biomass accumulation at 48-h incubation. These designs were set up for 5 or 4 factors for PBSD and FFSD, respectively (see Results and Discussion) with three coded levels (−1, 0, +1), to evaluate their linear effects, as well as the interaction effects among variables (FFSD) on cell growth. Three repetitions of the center point were run for each experiment. The results were fitted with a first-order model, estimating the coefficient (slope) for each factor and its level of significance (Strobel and Sullivan 1999; Montgomery 2001).

Box–Behnken designs (BBD, Box and Behnken 1960) set up for three factors at three coded levels (−1, 0, +1) were run mainly to evaluate the quadratic effects and two-way interactions among the variables and to construct a second order polynomial model. The variable ranges were selected according to the PBSD results, and fours repetitions of the center point were run in order to determine the experimental error.

In both screening and BBD designs the variable levels*X*_*i*_ were coded as*x*_*i*_ according to equation (1):


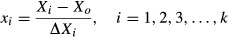
(1)

where*x*_*i*_ and*X*_*i*_ are the dimensionless codified value and the actual value of an independent variable, respectively,*X*_0_ is the real value of an independent variable at center point and Δ*X*_*i*_ the step change.

Upper and lower limits of the variables*X*_1_,*X*_2_, and*X*_3_ of the BBD are shown in Table2 (see Results and Discussion). The response surface methodology (RSM) was used to analyze this experimental design. The second-degree model used to fit the response to the independent variables is shown in equation (2):


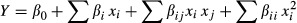
(2)

where*Y* is the predicted response,*x*_*i*_,*x*_*j*_ are the input variables that influence the response variable (*Y*),*β*_0_ is the intercept,*β*_*i*_ is the*i*th linear coefficient,*β*_*ii*_ is the*i*th quadratic coefficient, and*β*_*ij*_ is the*ij*th interaction coefficient.

**Table 2:** Box–Behnken optimization design and measured response.

Trial no.	Factor			Response OD_600_^1^
	*X*_1_/(*x*_1_)	*X*_2_/(*x*_2_)	*X*_3_/(*x*_3_)	
1	5/(+1)^2^	10/(+1)	42/(0)	26.70
2	2.5/(0)	5/(0)	42/(0)	24.70
3	5/(+1)	5/(0)	60/(+1)	35.60
4	2.5/(0)	0/(−1)	24/(−1)	10.20
5	2.5/(0)	5/(0)	42/(0)	23.20
6	2.5/(0)	10/(+1)	60/(+1)	33.30
7	0/(−1)	0/(−1)	42/(0)	10.50
8	2.5/(0)	0/(−1)	60/(+1)	21.20
9	2.5/(0)	5/(0)	42/(0)	20.50
10	0/(−1)	5/(0)	24/(−1)	12.10
11	0/(−1)	10/(+1)	42/(0)	16.20
12	2.5/(0)	10/(+1)	24/(−1)	13.75
13	5/(+1)	5/(0)	24/(−1)	14.65
14	5/(+1)	0/(−1)	42/(0)	11.40
15	2.5/(0)	5/(0)	42/(0)	24.00
16	0/(−1)	5/(0)	60/(+1)	22.20

*X*_1_ = (NH_4_)_2_SO_4_ (g·L^−1^);*X*_2_ = CMz (g·L^−1^);*X*_3_ = time (h).

1 Data represent the average ± SD of three independent experiment.

2 Numbers between brackets are the coded values of the variables.

Statistical and numerical analyses were carried out by analysis of variance (ANOVA) and multiple regression using the software Essential Regression (Experimental Design in MS Excel-free, user-friendly software package, v. 2003).

Responses measured were subjected to multiple regression analysis by least squares. Statistical significance of the regression coefficient was evaluated by Student's*t*-test. Adequacy of the mathematical models of the regression was assessed with Fisher's*F* test.

All experimental designs were randomized to exclude bias. Unless otherwise indicated, all experiments carried out in liquid media were performed in 250-mL Erlenmeyer flasks incubated at 28 ± 1°C at pH between pH 4.0 and 5.5 units and at an agitation rate of 160 rpm provided by an orbital shaker for the period of time indicated in each case. The flask:medium volume ratio was 5:1.

### Bioreactor assays

Batch fermentations were carried out in a 5-l New Brunswick FS300 baffled fermenter (New Brunswick Scientific Co., New Brunswick, NJ), with two flat-bladed turbines with six blades (Rushton turbine). The fermenter was equipped with controllers for pH, temperature, agitation, and dissolved oxygen concentration and contained 4.0-l of the optimized medium previously determined. Inocula were carried out essentially as described for Erlenmeyer flasks, except that the liquid medium for the inoculum was the same as for the fermenter. Agitation and aeration were varied to maintain dissolved oxygen concentration at 40% air saturation. Foam production was controlled by addition of antifoam (Antifoam 289; Sigma, Saint Louis, MO).

### Chymosin production

Yeast cells grown in the optimized culture medium were pelleted (5500*g* for 15 min) and washed three times with sterilized distilled water and finally suspended in the original volume of YNB medium at pH = 4.0 (0.10 mol/L CCB) containing methanol (10 mL·L^−1^), and sorbitol was added to attain a concentration of 50 g·L^−1^ (Celik et al. 2009). Thereafter, cultures were fed daily with methanol in 0.10 mol/L CCB pH = 4.0, to attain a methanol concentration of 10 mL·L^−1^. Methanol additions were carried out along a 4-day period to induce recombinant bovine prochymosin expression. Every 24 h, yeast cells of culture samples were spun down and crude supernatants were assayed for IMCU and protein content.

### Analytical determinations

For routine assays, biomass production was monitored by OD_600nm_ determinations in an Spectrum vis Spectrophotometer, after washing the cells thoroughly with phosphate buffer 0.10 mol/L (pH 6.5) and centrifugation at 5500*g* for 15 min. Biomass was also gravimetrically evaluated by determining the CDW (g·L^−1^) with a thermobalance Precisa XM50 (Precisa Gravimetrics AG, Dietikon, Switzerland) after washing the cells three times with distilled water as described above.

The following relationships were calculated from calibration curves between the two parameters:

1. CDW (g·L^−1^) = OD_600nm _× 0.45 g·L^−1^ for cells growing in glycerol.
2. CDW (g·L^−1^) = OD_600nm_ × 0.50 g·L^−1^ for cells growing in C sources other than glycerol.

For routine assays, the amount of RS was determined by a dinitrosalicylic acid assay with d-glucose standard solution. Then, the RS content was confirmed with an enzymatic kit that detects d-glucose and d-fructose (Boehringer Mannheim/R-Biopharm AG, Darmstadt, Germany, Cat. No. 10716260035). Glycerol was determined by the enzymatic kit Boehringer Mannheim/R-Biopharm, Cat. No. 10148270035.

Chymosin activity was assessed as International Milk Clotting Units (IMCU) in aliquots of cell-free supernatant of the inducing medium as described in Jiang et al. (2012). For a control, an aliquot of the culture supernatant was boiled at 100°C for 20 min before the enzyme assay. One unit of milk-clotting activity was defined as the quantity required for clotting 1 mL of skimmed milk in 40 min at 37°C. Commercial recombinant bovine chymosin (Maxiren® DSM; DSM, Heerlen, the Netherlands) was used as a standard reference for IMCU activity assays.

Total soluble protein in the culture supernatant was quantitated by the Bradford assay employing bovine serum albumin as standard (Bradford 1976).

All experiments described herein were carried out, at least, in duplicate and repeated, at least, twice.

## Results and Discussion

### Exploring the use of GRE as a carbon source

A variety of C sources were compared to GRE for the propagation of*K. pastoris* (Clone 1) biomass. Maximum biomass production (stationary phase of growth) was measured and results are tabulated in Table1. At comparable C source molar concentration, GRE was able to produce 11% higher biomass than 50.0 g·L^−1^ glycerol, which is a typical concentration recommended for*K. pastoris* growth (Sreekrishna and Kropp 1996). Media with d-glucose (40.0 g·L^−1^) was the best C source for biomass production, but the GRE at similar sugar concentration fell short of this production value by only 6%. Moreover, when costs of C-energy sources is considered, GRE appeared as a promising one, particularly when compared to typical ones used for the growth of*K. pastoris*, as pure d-glucose or glycerol. On the other hand, biomass yield values attained with GRE were comparable to all the C sources employed, being around 40%.

**Table 1:** Values of maximum specific growth rate (*μ*_max_), generation time (*T*), maximum cell dry weight (CDW_max_), and yield CDW/substrate (Y_CDW/S_) for*Komagataella pastoris* grown in YNB supplemented with different C sources.

C source	*μ*_max_ (h^−1^)	*T* (h)	CDW_max_ (g·L^−1^)	*Y*_CDW/S_
Glycerol (25.0 g·L^−1^)	0.202	3.4	13.1	0.42
Glycerol (50.0 g·L^−1^)	0.205	3.4	13.2	0.41
Glycerol (100.0 g·L^−1^)	0.206	3.4	14.0	0.40
Glycerol from biodiesel (25.0 g·L^−1^)	0.190	3.6	10.4	0.41
Glucose (40.0 g·L^−1^)	0.265	2.6	15.8	0.40
GRE (37.5 g·L^−1^)	0.149	4.7	14.8	0.39

GRE, grape pomace extracts.

All these results indicate that fermentable sugars from an agroindustrial waste as GRE could replace other commonly used fermentable and nonfermentable C sources assayed for biomass production of the genetically engineered*K. pastoris* strain. Similar behaviors were obtained when two other samples of grape pomaces were used. Although the specific growth rate in GRE was lower than those obtained in the presence of glucose or glycerol, the yields obtained (0.39 g·g^−1^) were comparable. It is noted that the cost of the C source is very important, and these grape wastes can be considered a*negative cost* C substrate, even when compared to other agroindustrial by-products tested,*v.gr*. biodiesel-derived glycerol. Moreover, GRE requires fewer purification steps when compared to biodiesel-derived glycerol.

### Selection of nitrogen and growth factors sources in the presence of GRE

Nitrogen is a main component of yeast biomass second to carbon, so an optimal N source for GRE-containing culture medium needs to be determined too. Comparative experiments carried out in YNB and in YNB w/o media using GRE as source of RS, showed that biomass production of the*K. pastoris* strain used was enhanced when a complete growing yeast medium (i.e., YNB) was employed. Thus, the addition of supplementary N source in the GRE-containing medium was studied.

**Figure 1:**
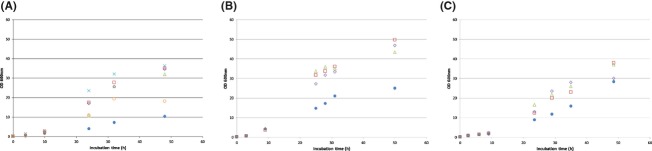
Effect of growth of*Komagataella pastoris* in GRE (A) with different N source: 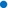 GRE without supplementation; 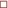 GRE + meat peptone, 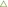 GRE + tryptone; 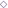 GRE + (NH_4_)_2_SO_4_; 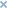 GRE + corn steep liquor; 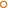 GRE + urea; (B) growth factors addition: 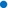 GRE without supplements; 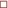 GRE + yeast extract; 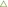 GRE + cane molasses; 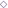 GRE + biotin; (C) at different pH values. 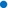 pH = 3, 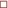 pH = 4, 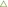 pH = 5, 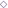 pH = 6. GRE, grape pomace extracts.

As Figure1A shows, yeast growth was enhanced when N (organic or inorganic) substrates were added. Because addition of the N sources used increased both maximal-specific growth rate and biomass concentration reached in the stationary phase of growth, GRE was considered deficient in N for Clone 1 growth. Curves depicted in Figure1 showed that the inorganic salt (NH_4_)_2_SO_4_ was as effective as meat peptone, tryptone, and CSL ones with respect to the highest biomass productions and productivities obtained at 48 h incubation.

Thus, for medium optimization studies, (NH_4_)_2_SO_4_ was selected mainly because of cost considerations, taking in mind that one of the main objective of this work was to lower the cost of the biomass production medium.

A common defined basal medium for*K*. (*=Pichia*)*pastoris* growth requires biotin as sole vitamin (Grasser et al. 2010). Moreover, Boze et al.(2001) and Ghosalkar et al. (2008) reported that supplementation with a vitamin mixture could improve*K. pastoris* growth. Thus, we assayed different vitamin supplementations of a GRE and (NH_4_)_2_SO_4_-containing medium. As Figure1B shows, addition of vitamins and growth factors increased yeast biomass production. It is also shown that the effect of biotin could be achieved by other complex vitamin sources like YE and the cheaper CMz. So, the use of low amounts of another industrial by-product as CMz was able to provide enough growth factors as biotin, avoiding the use of this expensive vitamin.

### Effect of pH on yeast growth

Growth at different pH values in the presence of GRE was analyzed to find the optimal range for biomass production of the recombinant*K. pastoris* strain. On the other hand, as chymosin production by this strain is strongly dependent on the pH of the induction medium (Noseda et al. 2013), the selection of an appropriate value for biomass production has to be taken into account in order to maintain a constant pH along the chymosin production bioprocess. As Figure1C shows, the optimal pH values were found to be between pH 4.0 and pH 5.0 units, meanwhile the extreme pH values tested (3.0 and 6.0 units) resulted in lower biomass productions. As chymosin induction occured optimally at pH 4.0 (Noseda et al. 2013), pH adjustment would not be necessary to induce enzyme production.

### DoEs

#### Screening designs

To study the feasibility of the use of GRE together with a BSM containing medium to produce biomass of*K. pastoris*, statistically designed experiments were conducted. A rich N source as meat peptone was used to avoid nutrient shortage during yeast propagation. With this aim, a FFSD was carried out with four variables with ranges (in L^−1^) from 0 to 63.5 and 5.0 g for RS from GRE and meat peptone, respectively, 0.0 to 2.0 mL of BSM (10×, Sreekrishna and Kropp 1996), and incubation time ranging from 24 h to 60 h. The results obtained were not statistically significant, but suggested that BSM negatively affected the growth of the yeast strain studied in both its linear term (−1.176) as in its interaction with GRE and incubation time (−1.518 and −1.337, respectively). Taking these and previous results (data not shown) into account, where BSM in the presence of GRE consistently negatively affected the growth of the*K. pastoris* strain used, it was excluded in the following experiments. This fact is notable because it is unnecessary to support a GRE growth medium with a complex salt mixture, thus simplifying the preparation even more.

PBSD were applied in order to screen the linear effects of culture variables that could significantly affect yeast biomass production. To do this, some of the main components of an universal complete yeast growth medium such as YNB were separately studied to see their influences on yeast biomass production of the transformed strain used, in the presence of GRE (67.0 g·L^−1^ RS). Five variables from YNB medium (a complete yeast growth medium) were studied:*X*_1_ (0.5–1.0 g·L^−1^) corresponded to a mixture of l-histidine, dl-methionine, and dl-tryptophan, being the final concentrations of 100, 200, and 200 mg·L^−1^, respectively, in the cultivation medium for the upper level (+1); (NH_4_)_2_SO_4_ (*X*_2_, 0.0–5.0 g·L^−1^) was included in order to confirm the need of a N source supplementation when GRE is used as C source; the macronutrients corresponded to the salts present in YNB medium (*X*_3_, 0.0–1.0 g·L^−1^) and were added to investigate if they inhibited the yeast growth as BSM did; micronutrients as PTM1 (*X*_4_, 0.0–2.0 g·L^−1^) and vitamin supplementation provided by YE (*X*_5_, 0.0–3.0 g·L^−1^) were also included. The results were fitted with a first-order model, estimating the coefficient (slope) for each factor and its level of significance (Strobel and Sullivan 1999) and the equation obtained for cell growth after 48 h incubation was:


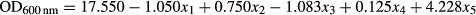
(3)

where*x*_*i*_ represent the codified values for the variables studied (amino acids mixture, (NH_4_)_2_SO_4_, macronutrients, PTM1 (Sreekrishna and Kropp 1996), and vitamins [YE], respectively). The*P* values for regression coefficients in bold characters were significant at 0.05 level of confidence.

**Figure 2:**
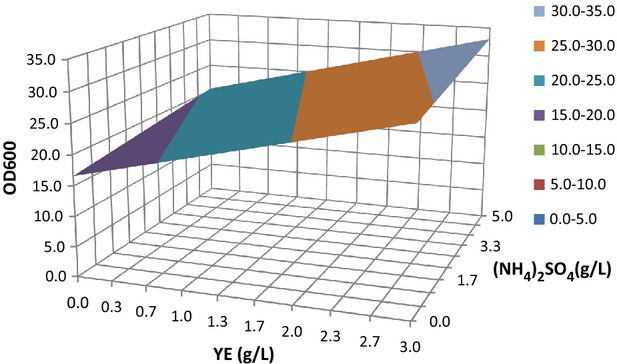
Surface plot for biomass production expressed as a function of (NH_4_)_2_SO_4_ and yeast extract (YE) concentrations for the PBSD screening, at level = 0 for the other variables. PBSD, Plackett–Burman screening designs.

So, the only variables influencing the growth of this*K. pastoris* strain positively and significantly were the N and vitamins sources (YE) (Fig.2). On the other hand, the lack of fit of the regression model was not significant, and the parameter*F*_significative_ (<10^−3^ <0.05) demonstrated a high significance for the regression. At the same time, the*F* value (19.08) was higher than the critical value (3.48) obtained from tables for a significance level 95% with 5 and 9 degrees of freedom. The quality of fit was determined with the determination coefficient*R*^2^ (= 0.914).

#### Box–Behnken optimization design

As any bioprocess, biomass production is a multivariable one, so variables interactions must be taken into account. Thus, after the main culture conditions were selected using statistical screening designs as PBSD and FFSD, a BBD was employed to reach the optimized growth parameters. It included only three variables because the effectiveness of BBD together with RSM is greater when fewer than five factors are considered (Kennedy and Krouse 1999). Among the optimization designs, BBD are very convenient because they use a reduced number of experimental runs to generate a predictive model compared to either full factorial or central composite designs (Montgomery 2001). The most interesting property of BBD is that each factor takes only three levels instead of the five required for central composite designs (unless*α* = 1), is experimentally more convenient, and also less expensive to run with the same number of factors. Finally, BBD ensures that all factors are never set at their high levels simultaneously (Box and Behnken 1960; Bodea and Leucuta 1998; Ragonese et al. 2002; Bae and Shoda 2005; Cui et al. 2012) and RSM analysis allowed the fine-tuning of the most significant industrially productive variables when the ultimate goal is its application at a larger scale.

For all these reasons, a BBD was herein used to optimize the values of the main culture conditions to produce maximal yeast biomass: (NH_4_)_2_SO_4_ concentration, as it demonstrated that was as good as other N sources in the presence of GRE; CMz replacing YE as source of vitamins specially biotin, and incubation time.

Table2 shows the actual and coded levels of the three variables in the experimental design, and the response obtained corresponding to yeast biomass production expressed in OD_600nm_ units. After multiple regression analysis the regression coefficients*x*_1_*x*_1_ and*x*_2_*x*_2_ were statistically significant (*P* < 0.05) and their negative values suggest that N source and the vitamins have been maxed out (Table3). Also, all the interactions had significant and positive influence on biomass production in the ranges studied, indicating how much the slope with respect to one factor increases with increasing another factor (Kennedy and Krouse 1999).

**Table 3:** Statistical analysis of the optimization design for biomass production of*Komagataella pastoris*.

Coefficient	Value	SE	P*	*t*
*β*_0_	7.897	5.271	0.185	1.498
*x*_1_	0.026	1.092	0.982	0.024
*x*_2_	1.124	0.546	0.085	2.059
*x*_3_	−0.030	0.226	0.900	−0.131
***x***_**1**_***x***_**1**_	−**0.430**	**0.132**	**0.017**	−**3.257**
***x***_**2**_***x***_**2**_	−**0.169**	**0.033**	**0.002**	−**5.106**
x_3_*x*_3_	0.002	0.003	0.413	0.879
***x***_**1**_***x***_**2**_	**0.192**	**0.066**	**0.027**	**2.909**
***x***_**1**_***x***_**3**_	**0.060**	**0.018**	**0.017**	**3.288**
***x***_**2**_***x***_**3**_	**0.024**	**0.009**	**0.041**	**2.591**

*x*_1_ = (NH_4_)_2_SO_4;_*x*_2_ = CMz;*x*_3_ = time.

* The*P* values for regression coefficients in bold characters are significant at*P* < 0.05.

The maximal cell concentration obtained (OD_600nm_ = 35.60 units, or 17.80 g CDW·L^−1^) corresponded to the combination of coded levels of +1, 0, and +1 at 5.0 g·L^−1^ of (NH_4_)_2_SO_4_ and CMz with incubation time of 60 h.

**Figure 3:**
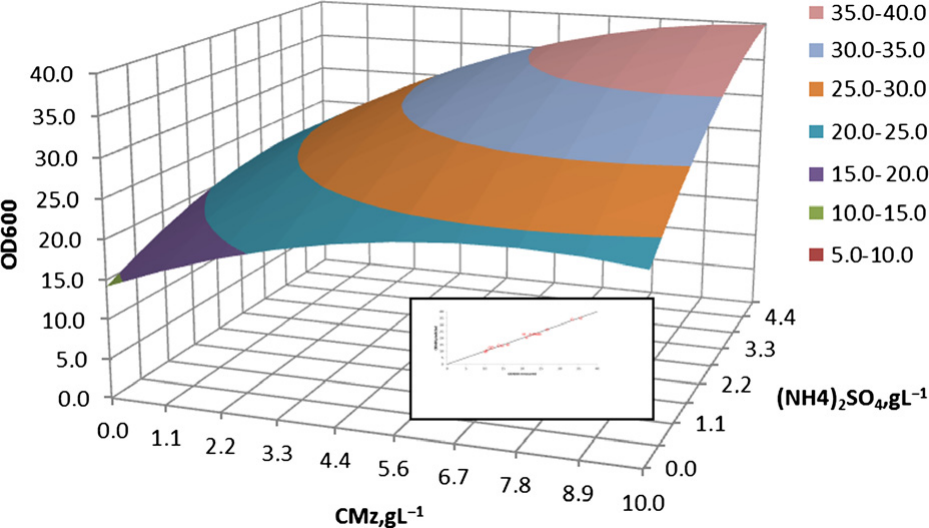
Response surface plot showing the effect of cane molasses (CMz) and (NH_4_)_2_SO_4_ concentrations at 60-h incubation time (+1) on biomass production of*Komagataella pastoris*.*Inset*: predicted versus experimental values for biomass expressed as OD_600nm_ units. The solid line depicted represents*y* *= x*.

Using the RSM, it was possible to identify the optimum region. Figure3 shows the 3D surface plot described by the regression model taking into account the influence of CMz (replacing YE as a source of vitamins according to Fig.2) and (NH_4_)_2_SO_4_ concentrations, at a constant coded level +1(=60 h) for incubation time. This plot shows that in the presence of maximal GRE concentrations, optimal biomass production could be obtained at the highest levels (+1) of both (NH_4_)_2_SO_4_ and CMz. Higher values of both CMz and (NH_4_)_2_SO_4_ were assayed, but no significant increase in biomass production was attained.

Concerning the goodness of fit, the determination coefficient value*R*^2^ = 0.981 indicated a high significance of the model and that only less than 2% of the response variations were not explained by the model (Haaland 1989).

ANOVA analysis was performed as well. The lack of fit of the regression model was not significant and the Fisher's*F* test (*P* < 0.05) demonstrated a high significance for the regression.

Another indicative proof of the high significance of the model obtained for yeast cell production is the parity plot representing predicted OD_600nm_ versus experimental values (Fig.3, inset). The plot was very close to*y *= *x*, and it could be assumed that there is satisfactory coincidence between experimental and predicted results.

### Validation of the model

Validation of the model was experimentally carried out in shake flask cultures using the optimized conditions reported. The validity of the mathematical model was proved by fitting the variables into the model equations, and then carrying out the experiments using the two extreme culture conditions found. From RSM, the model given in equation (2), and the regression coefficients of Table3, the combination of factors that maximize (1) or minimize (2) biomass production in the presence of 67 g·L^−1^ RS from GRE of the strain studied were:

1. 5.0 g·L^−1^ (NH_4_)_2_SO_4_, 10.0 g·L^−1^ CMz, and 60 h incubation. The model predicted for this combination a maximal OD_600nm_ = 39.9 (20.0 g·CDW·L^−1^) and an experimental average of 39.3 (19.6 g·CDW·L^−1^) was measured.
2. 0.0 g·L^−1^ (NH_4_)_2_SO_4_ and CMz, and 24 h incubation. The model predicted this will produce a minimum OD_600nm_ = 8.5 (4.3 g·CDW·L^−1^) and an experimental value of 9.9 (4.95 g·CDW·L^−1^) was measured.

So, an excellent correlation between predicted and experimental values was obtained. The results from these experiments indeed validate the mathematical model according to the absolute values of the standardized residues that were within the acceptable limits (data not shown). Thus, the strategy employed in this work as a whole proved to be adequate for the design and optimization of the bioprocess studied.

Furthermore, the optimized culture conditions obtained in Erlenmeyer flasks were assayed in a stirred tank reactor, with a working volume of 4 L. The volumetric production of biomass obtained after 36 h fermentation was 22.3 g·CDW·L^−1^.

### Chymosin expression

The*K. pastoris* recombinant strain was grown in the statistically optimized growth medium to further confirm chymosin production. The yeast cells cultured for 60 h in this GRE containing medium were harvested when CDW reached 20 g·L^−1^, a concentration recommended for the Mut+ strain to achieve an optimal heterologous protein yield in the fed-batch step on methanol (Sreekrishna and Kropp 1996), and then were treated as described in Materials and Methods to induce chymosin production. Chymosin and protein concentrations were measured in the supernatant. Chymosin reached values of 1.48·IMCU·mL^−1^ with a specific activity of 8.53·IMCU·mg^−1^ protein at 96 h post induction. A commercial chymosin assayed in our laboratory presented a specific activity of 19.05·IMCU·mg^−1^ protein, just 2.2 times higher than the maximal values obtained here. The chymosin production values obtained in Erlenmeyer flasks here were in accordance to the productions reported by Noseda et al. (2013) at this scale in different growth conditions.

## Conclusions

RSM was used to get the values of the main culture condition variables to produce maximal biomass of the recombinant*K. pastoris* strain constructed in our laboratory. RSM analysis allowed the fine-tuning of the most significant industrially productive variables.

The optimized growth medium proposed presented several advantages with respect to the traditionally culture media employed for this purpose; it is easy to prepare, and is much less expensive than those with the classical C sources used. The addition of (NH_4_)_2_SO_4_ and low concentrations of CMz was adequate for supplementing GRE deficiencies in nitrogen and growth factors as biotin for supporting the growth of the strain studied. On the other hand, other macro- and micronutrients, some of them representing a drawback when used in classical culture media, demonstrated to be unnecessary to support growth of this*K. pastoris* strain in GRE medium. Indeed, salts that are normally included in universal*K. pastoris* growth media such as BSM actually appeared to inhibit cells growth under our propagation conditions.

Additionally, the yeast growth medium presented herein makes use of by-products of the wine industry and sugar refineries to obtain biomass of a yeast producing chymosin, an enzyme of high commercial value. Particulary, the use of grape skin residues also reduces waste removal costs.

These results also enable two main issues with industrial implications. First, the considerable production of recombinant chymosin by yeast cells grown in the optimized culture medium in Erlenmeyer flasks, and thereafter scaled in 5-L agitated bioreactor, was similar to that obtained in our laboratory using a more complex medium (Cereghino et al. 2002), and comparable to a commercial chymosin brand. Second, the yeast biomass produced in high-cell-density cultivation with this simple and cheap medium, could also be employed to produce another value added product such as yeast autolysate to be used as flavorings or flavor enhancers in the food industry. At this respect, experiments are now being carried on in our laboratory.
